# The impact of the COVID-19 pandemic on the number of presentations of penetrating injuries to a UK major trauma centre

**DOI:** 10.1093/pubmed/fdab333

**Published:** 2021-08-25

**Authors:** Maria M Hickland, Philippa Massouh, Roxanne E Sutthakorn, Charlotte Greenslade, Cara Jennings, Fleur Cantle, Duncan Bew

**Affiliations:** GKT School of Medical Education, King’s College London, London SE1 1UL, UK; GKT School of Medical Education, King’s College London, London SE1 1UL, UK; GKT School of Medical Education, King’s College London, London SE1 1UL, UK; GKT School of Medical Education, King’s College London, London SE1 1UL, UK; Department of Emergency Medicine, King’s College Hospital NHS Foundation Trust, London SE5 9RS, UK; Department of Emergency Medicine, King’s College Hospital NHS Foundation Trust, London SE5 9RS, UK; Department of Trauma and Acute Surgery, King’s College Hospital NHS Foundation Trust, London SE5 9RS, UK

**Keywords:** emergency care, public health, violence

## Abstract

**Background:**

Knife-related violence is of growing concern in the UK. This study aims to investigate the impact of the COVID-19 pandemic on the frequency of penetrating injuries at a UK major trauma centre.

**Methods:**

This was a retrospective study comparing the number of patients attending the emergency department of King’s College Hospital (KCH) with a penetrating injury (gunshot or stab wound) during the ‘pandemic year’ (1 March 2020–28 February 2021) compared with the equivalent time period in the previous year. Penetrating injuries as a result of self-harm were excluded. The primary outcome was to assess whether there were any changes to the frequency of presentations during three periods of national lockdowns.

**Results:**

Lockdown 1 showed a 48.45% reduction in presentations in the ‘pandemic year’ compared to the previous year, lockdown 2 showed a 31.25% reduction; however, lockdown 3 showed an 8.89% increase in the number of presentations.

**Conclusion:**

Our findings suggest that despite the initial reduction in the number of presentations of penetrating injury during lockdown 1, this returned to normal levels by lockdown 3. Further research is required to understand the effects of government-imposed restrictions on interpersonal violence and identify appropriate methods of outreach prevention during a pandemic.

## Introduction

Knife-related violence is a growing problem in the UK. Between March 2011 and June 2019, the police recorded a 44% increase in the number of offences involving a knife or sharp object. As the site of 32% of recorded offences in 2019,^1^ London currently has the highest rates of knife crime in England and Wales.

On Monday 23 March 2020, the UK entered its first national lockdown, sanctioned by the government in order to curtail the spread of the COVID-19 virus in the country.^2^ Citizens were told to stay and work from home as much as possible to minimise social contact to just members of their household and to only go out for essential trips such as to get food or medical provisions, to access healthcare or for 1 hour of exercise per day. Restrictions were lifted to some degree on 4 July 2020. During the following 12 months, the UK population lived under varying levels of social and travel restriction, with two further national lockdowns imposed from 5 November to 2 December 2020 and again from 6 January 2021 until 12 April 2021. Schools closed from 20 March 2020, with children of key workers still allowed to attend, and partially reopened on 1 June 2020. Schools remained open during the second national lockdown and closed again from 5 January 2021 until 8 March 2021.

A study carried out at KCH, London, found that presentations of injury due to penetrating trauma had dropped by 35% in the initial 5 weeks of the first national lockdown in 2020.^3^ Anecdotal evidence from the hospital’s emergency department (ED) doctors suggested that the frequency of presentations due to penetrating injury from interpersonal violence (IPV) decreased in the first national lockdown of 2020 but remained at normal levels during subsequent lockdowns.

This study aims to evaluate the effect of the COVID-19 pandemic on the frequency of presentations of weapon-assisted penetrating injuries to a major trauma centre and to deduce whether patterns emerge according to national lockdown dates.

## Methods

This is a retrospective observational study. The patient database was searched to identify patients who had attended the ED at KCH, London), with a penetrating injury, defined as gunshot or wound inflicted with a sharp object, between 1 March 2019 and 28 February 2021, inclusive. Penetrating injuries recorded as deliberate self-harm were excluded. Demographic data were obtained from electronic patient notes. Date of attendance, information surrounding the event, such as weapon used; alleged assailant and location of assault was also collected, as well as whether patient had previously attended with a penetrating injury due to IPV and whether the patient was previously known to social services or Redthread, a youth violence intervention charity.

Data were then evaluated to compare the number of attendances for the control year (1 March 2019 to 29 February 2020), with the ‘pandemic’ year (1 March 2020 to 28 February 2021). The primary outcome was to assess whether there were any changes to raw figures during the control year and the ‘pandemic’ year, during the three periods of national lockdown. Dates used to compare lockdown periods over the 2 years were as follows: ‘Lockdown 1’ (23 March to 3 July); ‘Lockdown 2’ (5 November to 1 December); ‘Lockdown 3’ (21 December to the end of our data set −28 February).

Patients’ home postcodes were used to calculate the lower super output area, which in turn was used to identify the multiple index of deprivation (IMD) using the English indices of deprivation 2019. IMD could not be calculated if the patient’s postcode was not from an English address, if it was missing from the patient’s notes or if the patient did not have a fixed abode.

Statistical analysis was carried out using SPSS Statistics Version 27. *P* values were calculated using a chi-square test where data were categorical. A Kolmogorov–Smirnov test of normality showed that data for age were not normally distributed; therefore, a Mann–Whitney U test was used to calculate a *P* value. Statistical significance was set at a value of *P* < 0.05.

## Results

During the study period, 609 patients with penetrating injuries were identified from the database (569 sharp injuries, 40 firearm injuries). A total of 35 cases were excluded due to factors such as incorrect coding (i.e. not being a penetrating injury); insufficient information e.g. patient left the department without being seen; or if patients presented to the ED with an existing injury or for removal of sutures. After excluding these patients, 574 underwent further analysis. There were 299 presentations during the control year and 275 presentations during the ‘pandemic’ year. The demographics of these patients are shown in Table 1.

**Table 1:** Demographics of patients attending KCH’s ED for penetrating injuries due to interpersonal violence

	ALL N = 574		March 2019–February 2020 N = 299		March 2020–February 2021 N = 275		*P* value
Age, years, mean (SD)	28.4 (12.3)		28.6 (12.7)		28.2 (11.9)		0.873
Gender Male Female Unspecified	540 33 1	94% 6% 0.2%	288 11 0	96% 4% 0%	252 22 1	92% 8% 0.4%	0.048
Ethnicity Asian—Chinese Asian—Pakistani Asian—Indian Asian—Other Black African Black British Black Caribbean Black other Mixed white and black African Mixed white and black Caribbean Mixed other White British White Irish White Other Other ethnic group Unknown	1 2 5 7 69 106 51 26 8 5 8 106 4 33 33 110	0.4% 0.4% 0.8% 1.2% 12% 18.4% 8.9% 4.5% 1.4% 0.9% 1.4% 18.4% 0.7% 5.7% 5.7% 19.2%	0 1 3 4 35 65 23 11 5 0 5 72 1 18 19 37	0% 0.3% 1% 1.3% 11.7% 21.7% 7.7% 3.7% 1.7% 0% 1.7% 24.1% 0.3% 6% 6.4% 12.4%	1 1 2 3 34 41 28 15 3 5 3 34 3 15 14 73	0.4% 0.4% 0.7% 1.1% 12.4% 14.9% 10.2% 5.4% 1.1% 1.8% 1.1% 12.4% 1.1% 5.4% 5.1% 26.5%	<0.001
Deprivation decile 1 2 3 4 5 6 7 8 9 10 No fixed abode Postcode unknown	26 125 156 90 51 33 21 20 5 6 14 27	4.5% 21.8% 27.2% 15.7% 8.9% 5.7% 3.7% 3.5% 0.9% 1.0% 2.4% 4.7%	18 57 81 52 31 11 10 7 4 3 12 13	6.0% 19.1% 27.1% 17.4% 10.4% 3.7% 3.3% 2.3% 1.3% 1.0% 4.0% 4.4%	8 68 75 38 20 22 11 13 1 3 2 14	2.9% 24.7% 27.3% 13.8% 7.2% 8.0% 4.0% 4.7% 0.4% 1.1% 0.7% 5.1%	0.057
Weapon used Knife Glass bottle Unknown weapon—stab wound Screwdriver Multiple weapon types Other weapon Gun	297 12 199 6 11 12 37	51.7% 2.1% 34.7% 1% 1.9% 2.1% 6.4%	145 6 120 3 5 6 14	48.5% 2% 40.1% 1% 1.7% 2% 4.7%	152 6 79 3 6 6 23	55.3% 2.2% 28.7% 1.1% 2.2% 2.2% 8.4%	0.129
Alleged assailant Self Partner Unknown to patient Acquaintance Not disclosed Family member	11 22 141 59 313 28	1.9% 3.8% 24.6% 10.3% 54.5% 4.9%	7 12 62 33 173 12	2.3% 4.0% 20.8% 11.0% 57.9% 4.0%	4 10 79 26 140 16	1.6% 3.4% 28.9% 9.6% 51.0% 5.5%	0.225
Previously known to Social Services	98	17%	55	18%	43	16%	0.391
Previously attended due to interpersonal violence	99	17%	52	17%	47	17%	0.924
Previously known to Redthread	19	3.3%	10	3.3%	9	3.3%	0.969
Previously attended due to penetrating injury	63	11%	37	12%	26	9.0%	0.264

**Fig. 1:**
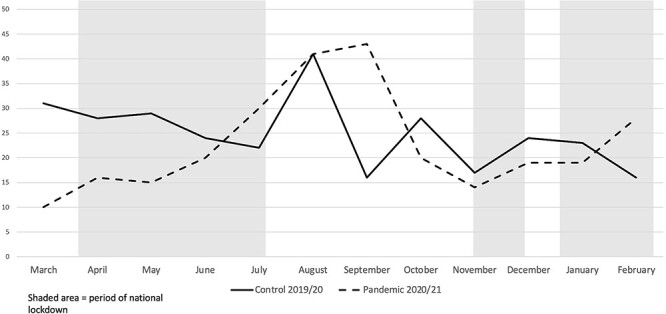
The number of patients presenting to KCH’s ED with penetrating injuries in the control year versus the pandemic year.

Presentations to the ED during each lockdown period can be found in Table 2. The first lockdown has the largest reduction in comparison with the previous year, the second shows a small decrease and the third a small increase in numbers. Lockdown period 1 in 2019 had 101 presentations compared with 54 in 2020 for the same period, showing a 46.53% decrease. Lockdown period 2 had 16 presentations compared to 11 over the same period in 2020, showing a 31.25% decrease. Lockdown period 3 had 49 presentations compared to 46 in the same period in 2019, showing 6.52% increase. This trend is highlighted in Fig. 1.

**Table 2:** Number of patients attending KCH’s ED for penetrating injuries as a result of interpersonal violence in the three periods of national lockdowns compared to the equivalent time period in the previous year

Lockdown period	Dates analysed	Number of presentations	Percentage change 2019 to 2020
Lockdown 1	23 March–3 July 2019	101	−46.53
	23 March–3 July 2020	54	
Lockdown 2	5 November–1 December 2019	16	−31.25
	5 November–1 December 2020	11	
Lockdown 3	21 December 2019–29 February 2020	46	+6.52
	21 December 2020–28 February 2021	49	

## Discussion

### Main finding of this study

This study’s main finding was that the number of presentations due to penetrating injury decreased during the first national lockdown of 2020. The two subsequent national lockdowns in 2020 did not bring about another decrease in presentations, however, with these staying at similar levels as the same period the year before. The demographic of patients presenting to the ED during the ‘pandemic’ year changed. There were significantly more females presenting during the ‘pandemic’ year compared to the control year (8% versus 4%). There was also a statistically significant difference in ethnicity between the 2 years, noticeably with an increase where the ethnicity of the patient was unknown and a decrease in white British and black British patients. There were no significant differences between the 2 years for age, deprivation decile, previously known to social services or Redthread, previous attendances due to penetrating injury or IPV, alleged assailant and weapon used.

Cohen and Felson’s Routine Activity Theory states that crime is most likely to happen where three elements regularly intersect: (i) a motivated offender, (ii) a suitable target and (iii) the absence of a capable guardian (such as security or police).^4^ According to two YouGov polls, there was a higher level of fear around catching COVID-19 in March 2020 than there was in July 2020, with 60% of people ‘very or somewhat scared’ about catching COVID-19 compared to 48%, respectively. People also reported being more likely to change their behaviour to prevent contracting the virus with 80% of polled people saying they would avoid public places in March compared to 60% in July.^5^ This may have reduced the number of offenders or targets from streets early on in the pandemic. Combined with a larger police presence, the closing of schools, pubs, restaurants and shops during the first national lockdown, it is therefore no surprise that there was a decrease in penetrating injury presenting to the ED.

In Autumn of 2020, during the buildup to the second wave of the COVID-19 pandemic in the UK when widespread vaccination had not yet been rolled out and the first COVID-19 variant of concern (the so-called ‘Kent variant’) was spreading throughout the South-East of the country, neither the fear of catching COVID-19, nor an avoidance of public places reached the same levels as at the beginning of the pandemic.^5^^,^^6^ Although studies show that adherence to social distancing measures remained high throughout lockdown periods, the lack of decline in ED presentations with penetrating injury during the second and third UK lockdowns show that the pandemic was no longer a factor in reducing these injuries.^7^

### What is already known on this topic

In the UK, two studies saw a decrease in the number of presentations to the ED due to penetrating injury in the first 5 and 10 weeks of the pandemic, respectively.^3^^,^^8^ However, one study showed that stabbings remained statistically stable during the first 7 weeks of the first UK lockdown compared to 2019.^9^

The drop in admissions due to penetrating injury seemed to be echoed across England during the first wave of the pandemic. A report by the Office for National Statistics shows admissions due to assault with a sharp object decreased by 23% between April and July 2020 compared to the same period the year before, which the report attributes to less social interaction due to lockdown measures.^10^ A decrease of 27% in hospital admissions for assault with a sharp object was seen in Greater London over the same time period.^11^

Anecdotally, KCH’s ED staff had noticed no significant drop in trauma presentations during the second and third lockdowns of 2020 a pattern, which was seen in our study. Interestingly, however, NHS data show that there was a decrease in hospital admissions for assault by a sharp object of 31.6% in Greater London in November and December 2020 compared to the same months in 2019.^12^ The difference in injury pattern may be due to this paper only covering one centre which may not be representative of London or the UK as a whole.

Looking outside of the UK, there are studies that show either an increase in penetrating injury presentations during their country’s first lockdown or a stable number.^13–16^ Waseem *et al*.’s^17^ literature review show no definitive pattern in trauma caused by assault during lockdown dates according to 13 papers from seven countries. Ultimately showing that the correlation between penetrating injury and national or local lockdowns found in this paper were not seen globally across major trauma centres.

The demographics of patients attending the ED with penetrating injuries are similar to what has previously been shown in other studies. The majority of patients attending with these injuries in our study were male (94%), in keeping with previous data.^18^^,^^19^

A previous retrospective cohort study carried out in a different London-based ED looking at the epidemiology of stab wounds among young people over a 10-year period found that over 50% of patients suffering knife wounds were under the age of 25.^19^ Furthermore, a large proportion (71%) of the patients were from the most deprived socioeconomic quintile, compared with just 1% from the least deprived quintile. Our study corroborates these data as the mean age of patients in our study was 28.4 years with 54% being 25 and under. We also observed an association with deprivation with more than half of patients falling into the top 3 most deprived deciles.

### What this study adds

This study is a partly a continuation of that conducted by Olding *et al*.^3^ as it analyses the pattern of patient presentations throughout the first national lockdown at the same major trauma centre. Although it does not include episodes of self-harm, this study does show the trend of a lower number of presentations due to assault with a sharp object over the first national lockdown in 2020, compared to the same period of 2019. This study then extrapolates further by looking at presentation frequency during subsequent national lockdowns and is, to date, the only study including a full year’s worth of patient presentations from the beginning of the COVID-19 pandemic. As the COVID-19 pandemic continues across the world, the patterns found in this study show that future lockdowns may not bring a decrease in penetrating injury to the ED, and services should be prepared as such. Also, due to the high number of patients in this study under the age of 25, it is vitally important that charities and government schemes aiming to prevent IPV among young people are still able to function during the pandemic and any future national or regional lockdowns as young people are still vulnerable.

### Limitations of this study

Although this is the first paper to analyse a year’s worth of data, not all presentations for the entirety of the UK’s third lockdown have been collected and analysed to look for increase in presentations as social restrictions are lifted, as seen at the end of the first national lockdown. In addition, this is a retrospective assessment of one trauma centre. So, while the results found here do concur somewhat with other British studies, but they may not reflect patterns seen in London or the UK as a whole. Patient records did not always contain all the demographic information this study aimed to collect.

Data surrounding the details of the assault were not always easy to retrieve. Most commonly, the alleged assailant was not disclosed, the location of the assault was not accurately recorded, and a large proportion of the weapons used was unknown. This reflects the difficulty clinicians have on the ground when understanding the nature of the event with limited details.

### Data sharing

The data for this article were provided by KCH NHS Foundation Trust with permission. Data will be provided on request after communication with the corresponding author and with the permission of the KCH NHS Foundation Trust.

### Conflicts of interest

There are no conflicts of interest to declare.

## Notes

**Maria M Hickland**, Medical Student

**Philippa Massouh**, Medical Student

**Roxanne E. Sutthakorn**, Medical Student

**Charlotte Greenslade**, Medical Student

**Cara Jennings**, Emergency Medicine Consultant

**Fleur Cantle**, Emergency Medicine Consultant

**Duncan Bew**, Trauma and Acute Surgery Consultant
